# Framingham Heart Study 100K project: genome-wide associations for cardiovascular disease outcomes

**DOI:** 10.1186/1471-2350-8-S1-S5

**Published:** 2007-09-19

**Authors:** Martin G Larson, Larry D Atwood, Emelia J Benjamin, L Adrienne Cupples, Ralph B D'Agostino, Caroline S Fox, Diddahally R Govindaraju, Chao-Yu Guo, Nancy L Heard-Costa, Shih-Jen Hwang, Joanne M Murabito, Christopher Newton-Cheh, Christopher J O'Donnell, Sudha Seshadri, Ramachandran S Vasan, Thomas J Wang, Philip A Wolf, Daniel Levy

**Affiliations:** 1 The National Heart, Lung, and Blood Institute's Framingham Heart Study, Framingham, MA, USA; 2 Department of Mathematics and Statistics, Boston University, Boston, MA, USA; 3 Boston University School of Medicine, Boston, MA, USA; 4 Whitaker Cardiovascular Institute, Boston University School of Medicine, Boston, MA, USA; 5 Department of Biostatistics, Boston University School of Public Health, Boston, MA, USA; 6 Section of General Internal Medicine, Boston University School of Medicine, Boston, MA, USA; 7 Cardiology Division, Massachusetts General Hospital, Harvard Medical School, Boston, MA, USA; 8 Broad Institute of Harvard and MIT, Cambridge, MA, USA

## Abstract

**Background:**

Cardiovascular disease (CVD) and its most common manifestations – including coronary heart disease (CHD), stroke, heart failure (HF), and atrial fibrillation (AF) – are major causes of morbidity and mortality. In many industrialized countries, cardiovascular disease (CVD) claims more lives each year than any other disease. Heart disease and stroke are the first and third leading causes of death in the United States. Prior investigations have reported several single gene variants associated with CHD, stroke, HF, and AF. We report a community-based genome-wide association study of major CVD outcomes.

**Methods:**

In 1345 Framingham Heart Study participants from the largest 310 pedigrees (54% women, mean age 33 years at entry), we analyzed associations of 70,987 qualifying SNPs (Affymetrix 100K GeneChip) to four major CVD outcomes: major atherosclerotic CVD (n = 142; myocardial infarction, stroke, CHD death), major CHD (n = 118; myocardial infarction, CHD death), AF (n = 151), and HF (n = 73). Participants free of the condition at entry were included in proportional hazards models. We analyzed model-based deviance residuals using generalized estimating equations to test associations between SNP genotypes and traits in additive genetic models restricted to autosomal SNPs with minor allele frequency ≥0.10, genotype call rate ≥0.80, and Hardy-Weinberg equilibrium p-value ≥ 0.001.

**Results:**

Six associations yielded p < 10^-5^. The lowest p-values for each CVD trait were as follows: major CVD, rs499818, p = 6.6 × 10^-6^; major CHD, rs2549513, p = 9.7 × 10^-6^; AF, rs958546, p = 4.8 × 10^-6^; HF: rs740363, p = 8.8 × 10^-6^. Of note, we found associations of a 13 Kb region on chromosome 9p21 with major CVD (p 1.7 – 1.9 × 10^-5^) and major CHD (p 2.5 – 3.5 × 10^-4^) that confirm associations with CHD in two recently reported genome-wide association studies. Also, rs10501920 in *CNTN5 *was associated with AF (p = 9.4 × 10^-6^) and HF (p = 1.2 × 10^-4^). Complete results for these phenotypes can be found at the dbgap website http://www.ncbi.nlm.nih.gov/projects/gap/cgi-bin/study.cgi?id=phs000007.

**Conclusion:**

No association attained genome-wide significance, but several intriguing findings emerged. Notably, we replicated associations of chromosome 9p21 with major CVD. Additional studies are needed to validate these results. Finding genetic variants associated with CVD may point to novel disease pathways and identify potential targeted preventive therapies.

## Background

Cardiovascular disease (CVD) and its most common manifestations, coronary heart disease (CHD), stroke, heart failure (HF), and atrial fibrillation (AF) are major causes of morbidity and mortality. In many industrialized countries CVD claims more lives each year than any other disease. In the United States, for example, heart disease and stroke are the first and third leading causes of death [1]. At age 40 the lifetime risk of developing CHD is one in two for men and one in three for women [2], the lifetime risk for stroke is one in six for men and one in five for women [3], the lifetime risk for HF is one in five in men and women [4] and the lifetime risk for AF is one in four in both sexes [5].

Prior Framingham Heart Study research points to strong familial patterns of CVD, HF, and AF [6-8] and such evidence is consistent with a genetic effect. Several single gene variants associated with CHD and atherosclerotic CVD have been reported [9-13]. A substantial body of research has also identified a number of genetic variants associated with HF and AF [14,15].

We report results of a genome-wide association study of four CVD outcomes in community-based Framingham Heart Study participants who were enrolled without regard to disease status. Analysis for each specific outcome was restricted to those free of the condition at baseline. We also provide association results for previously reported candidate genes and candidate regions for these CVD outcomes.

## Methods

### Study sample

In 1948, 5209 men and women from Framingham, Massachusetts, who were between 28 and 62 years of age, were recruited to participate in the Framingham Heart Study [16]. Periodic clinic visits, performed every two years, included a medical history, physical examination focusing on the cardiovascular system, laboratory tests, and electrocardiogram. The offspring cohort of the Framingham Heart Study began in 1971, with the enrollment of 5124 offspring and spouses of offspring of original participants [17]. Repeated examinations of the offspring cohort occurred approximately every 4 years, except for an 8 year interval between their initial and second visit. At each clinic visit, participants gave written informed consent. The consent documents and the examination content were approved by the Institutional Review Board at Boston University Medical Center (Boston, Massachusetts).

### Phenotype definition & methods

All participants in both cohorts who were free of a specific condition at enrollment were analyzed for onset of that endpoint during follow up through the end of 2004. All suspected CVD events were reviewed and adjudicated by a panel of three Framingham physician investigators after review of all available Framingham Heart Study examination records, hospitalization records, and physician notes, using previously published criteria [18].

For these analyses, we considered four groups of events: major CHD events included recognized myocardial infarction, coronary insufficiency, and death due to CHD; major atherosclerotic CVD events included major CHD plus atherothrombotic stroke; the remaining groups were HF and AF. Myocardial infarction was diagnosed by the presence of 2 out of 3 clinical criteria: new diagnostic Q-waves on ECG, prolonged ischemic chest discomfort, and elevation of serum biomarkers of myocardial necrosis. CHD death was established upon review of all available records, if the cause of death was probably CHD and no other cause could be ascribed.

Atherothrombotic brain infarction was defined as a non-embolic acute-onset focal neurological deficit of vascular etiology that persisted for more than 24 hours or an ischemic infarct was documented at autopsy.

History of interim hospitalizations and symptoms of HF were obtained at each clinic examination; outside medical records were evaluated for participants who did not attend an examination. Three physicians reviewed all suspected interim events using Framingham Heart Study clinic notes, external physician reports and hospitalization records. HF was diagnosed when at least two major criteria were present, or one major and two minor criteria. Major criteria were paroxysmal nocturnal dyspnea, pulmonary rales, distended jugular veins, enlarging heart size on chest radiography, acute pulmonary edema, hepatojugular reflux, third heart sound, jugular venous pressure of 16 cm or greater, weight loss of 4.5 kg or greater in response to diuresis, pulmonary edema, visceral congestion, or cardiomegaly on autopsy. Minor criteria counted only if not attributed to another disease. Minor criteria were bilateral ankle edema, nocturnal cough, shortness of breath on ordinary exertion, hepatomegaly, pleural effusion, vital capacity decreased by one third from previous maximum, and heart rate ≥120 beats/min.

AF was diagnosed when, upon review by a study cardiologist, AF or atrial flutter was present on an ECG obtained from a routine Framingham clinic examination or from a hospital or physician record. HF was defined on the basis of review of medical records and the finding of concurrent presence of two major or one major plus two minor criteria [19].

### Genotyping methods

The accompanying Overview [20] provides details of the genotyping methods used in this investigation. The Affymetrix 100K chip with 112,990 autosomal SNPs was used to genotype individual participant DNA on the Framingham Heart Study family plate set. SNPs were excluded for minor allele frequency < 0.1 (n = 38062); call rate < 0.8 (n = 2346); Hardy Weinberg equilibrium p value < 0.001 (n = 1595). After these exclusions, 70,987 SNPs were available for analysis.

### Statistical methods

Proportional-hazards models were used to analyze time to each endpoint, stratified by cohort, using covariate values obtained at enrollment. Models were adjusted for (i) sex and age, or (ii) sex, age and multiple covariates. For CVD and CHD, covariates included smoking, diabetes, systolic BP, anti-hypertensive treatment and total cholesterol; for HF, covariates were smoking, diabetes, systolic BP, anti-hypertensive treatment and body mass index; for AF, covariates were diabetes, systolic BP, anti-hypertensive therapy and valve disease. Deviance residuals estimated from each model were standardized (mean 0, variance 1) to form the phenotypes analyzed with genetic models. For genotype-phenotype association analyses, we assumed an additive-allele model of inheritance and we conducted association tests using regression models with generalized estimating equations (GEE), as well as family-based association testing using FBAT. Due to relatively small numbers of outcome events and non-normality of the deviance residuals, we decided *a priori *not to perform linkage analysis on outcomes residuals. The distribution of observed p values for the four CVD outcomes was compared to that which would be expected under the null hypothesis of no genetic associations with outcomes.

### Candidate gene analyses

GEE and FBAT additive genetic effect models also were run for SNPs in or near candidate genes for each of the CVD outcomes. Candidate genes were selected after separate literature searches for each outcome. All SNPs across the interval extending from 200 Kb proximal to the start to 200 kb beyond the end of each gene were eligible if the minor allele frequency was ≥0.1, the genotype call rate was ≥0.8, and the Hardy-Weinberg equilibrium p value was ≥0.001.

## Results

Four primary phenotypes were analyzed: major atherosclerotic CVD (n = 142), major CHD (n = 118), HF (n = 73), and AF (n = 151). Covariates for each outcome are listed in Table 1. In this sample, deviance residuals from multivariable models generally had low heritability: HF, 0.023 (SE = 0.054); Major CVD, 0.036 (SE = 0.058), Major CHD, 0.085 (SE = 0.061); and AF, 0.135 (SE = 0.058).

**Table 1:** Phenotype definitions

Phenotype	Definition	Number of individuals	Number with event	Adjustment*
**Major CVD**	Myocardial infarction, coronary insufficiency, CHD death, or atherothrombotic stroke	1345	142	Age, sex; Multivariable: Age, sex, smoking, diabetes, systolic BP, anti-hypertensive therapy, total cholesterol
**Major CHD**	Myocardial infarction, coronary insufficiency, or CHD death	1345	118	Same as Major CVD
**Heart failure**	Heart failure, hospitalized or non-hospitalized	1345	73	Same as Major CVD except BMI added, total cholesterol removed
**Atrial fibrillation**	Atrial fibrillation or atrial flutter on ECG	1341	151	Age, sex; Multivariable: Age, sex, diabetes, systolic BP, anti-hypertensive therapy, valve disease

* Covariates in cohort-stratified proportional-hazards models for time to event

GEE additive genetic models yielded six associations with p values < 10^-5 ^and another 31 with p values < 10^-4 ^(see Table 2a for best 25). The lowest p-values for each CVD phenotype were as follows: major CVD, rs499818, p = 6.6 × 10^-6^; major CHD, rs2549513, p = 9.7 × 10^-6^; AF, rs958546, p = 4.8 × 10^-6^; HF: rs740363, p = 8.8 × 10^-6^. Of note, rs10501920 in *CNTN5 *was associated with AF (p = 9.4 × 10^-6^) and HF (p = 1.2 × 10^-4^). Three SNPs near *PHACTR1 *were associated with major CVD (rs499818, rs1512411, rs507369; lowest p = 6.6 × 10^-6^) and one of these was associated with major CHD (rs1512411; p = 6.3 × 10^-5^). Among GEE results for HF was rs939698 (p = 3.6 × 10^-4^) in *RYR2*, which has been implicated in arrhythmogenic right ventricular dysplasia/cardiomyopathy [21], a rare familiar cardiomyopathy.

**Table 2:** Additive Genetic Model – ordered by GEE (2a) and FBAT (2b) p-value Results

Phenotype	SNP	Chromosome	Position	GEE P value	FBAT P value	Gene
**2a. Results ordered by GEE p-value results**						

AF	rs958546	13	45,731,718	**4.78E-06**	0.104	
Major CVD	rs499818	6	13,440,446	**6.64E-06**	0.17	
AF	rs4776472	15	67,793,927	**7.87E-06**	0.042	
HF	rs740363	10	118,565,596	**8.82E-06**	0.065	*KIAA1598*
AF	rs10501920	11	98,998,383	**9.40E-06**	0.448	*CNTN5*
Major CHD	rs2549513	16	78,108,228	**9.65E-06**	0.106	
AF	rs10507539	13	45,732,707	**1.05E-05**	0.02	
Major CVD	rs1512411	6	13,439,076	**1.55E-05**	0.366	*PHACTR1, TBC1D7*
Major CVD	rs10511701	9	22,102,599	**1.67E-05**	0.132	
Major CVD	rs1556516	9	22,090,176	**1.86E-05**	0.071	
Major CVD	rs1537371	9	22,089,568	**1.87E-05**	0.068	
Major CHD	rs10497726	2	192,876,826	**1.98E-05**	0.046	*TMEFF2*
Major CHD	rs2962994	15	55,129,991	**1.98E-05**	0.279	*TCF12*
Major CHD	rs997651	17	61,344,845	**2.28E-05**	0.547	*MGC33887*
Major CVD	rs2148079	13	109,989,414	**2.33E-05**	0.026	*RAB20*
AF	rs10501918	11	98,971,412	**2.40E-05**	0.093	*CNTN5*
HF	rs10511633	9	17,151,527	**2.59E-05**	0.044	*C9orf39*
Major CHD	rs7836535	8	96,774,748	**2.63E-05**	0.003	
Major CHD	rs1820996	15	55,120,501	**2.83E-05**	0.218	*TCF12*
Major CHD	rs213168	15	55,028,949	**3.09E-05**	0.278	*TCF12*
Major CHD	rs997652	17	61,344,827	**3.22E-05**	0.613	*MGC33887*
AF	rs4590838	11	97,372,875	**4.03E-05**	0.248	
Major CHD	rs10516882	4	92,265,754	**4.33E-05**	0.858	
Major CVD	rs1742083	14	90,256,423	**5.23E-05**	0.138	*TTC7B*
**Major CVD**	rs507369	6	13,440,039	**6.23E-05**	0.137	*PHACTR1, TBC1D7*

**2b. Results Ordered by FBAT**						

Major CHD	rs10505879	12	22,539,123	0.058	**3.06E-05**	*KIAA0528*
Major CVD	rs39312	7	116,548,736	0.138	**4.37E-05**	*WNT2*
AF	rs10511311	3	113,538,529	0.003	**4.45E-05**	*CD200*
AF	rs1427828	12	88,264,967	0.018	**4.58E-05**	*DUSP6*
HF	rs10515869	5	163,444,804	0.029	**4.72E-05**	
AF	rs1751382	14	67,762,403	0.138	**5.14E-05**	*RAD51L1*
AF	rs1314913	14	67,769,347	0.126	**5.53E-05**	*RAD51L1*
AF	rs262467	6	120,497,469	0.117	**6.39E-05**	
AF	rs412253	4	31,119,019	0.086	**6.55E-05**	
Major CVD	rs39317	7	116,560,255	0.219	**6.72E-05**	*WNT2, ASZ1*
Major CVD	rs9886209	7	116,599,175	0.594	**6.95E-05**	*ASZ1*
Major CVD	rs10493900	1	98,357,234	0.801	**7.10E-05**	
AF	rs1298340	14	67,747,245	0.275	**7.40E-05**	*RAD51L1*
Major CVD	rs2452503	10	60,686,639	0.384	**9.94E-05**	*FAM13C1*
AF	rs324735	4	77,062,193	0.018	**9.98E-05**	
Major CHD	rs580069	11	121,794,555	0.074	**1.24E-04**	
AF	rs1604355	1	187,190,664	0.294	**1.29E-04**	*FAM5C*
Major CHD	rs559453	11	121,794,482	0.073	**1.32E-04**	
Major CHD	rs951442	15	31,705,234	0.003	**1.35E-04**	*RYR3*
HF	rs1176486	10	132,315,529	0.165	**1.49E-04**	
AF	rs2421954	2	63,665,926	0.003	**1.51E-04**	*LOC51057*
HF	rs9313999	5	163,444,569	0.015	**1.55E-04**	
AF	rs7676376	4	158,199,764	0.282	**1.72E-04**	PDGFC
Major CHD	rs10501127	11	33,698,233	0.251	**1.78E-04**	CD59
**AF**	rs1163397	3	110,400,929	0.002	**1.78E-04**	

Results of FBAT are provided in Table 2b. The lowest p values for each phenotype were: major CVD, rs39312 in *WNT2*, p = 4.4 × 10^-5^; major CHD, rs10505879, p = 3.1 × 10^-5^; AF, rs10511311 in *CD200*, p = 4.5 × 10^-5^; and HF, rs10515869, 4.72 × 10^-5^.

The distribution of observed GEE p values is presented in Table 3. Note that the ratio of observed to expected numbers is inflated only at very low p values.

**Table 3:** Distribution of Observed and Expected P Values from GEE models

P value group	Frequency	Percent	Expected*	Ratio**
0.10 ≤ p	254,464	89.6164	90.000%	1.00
0.01 ≤ p < 0.10	26,218	9.2334	9.000%	1.03
0.001 ≤ p < 0.01	2,892	1.0185	0.900%	1.13
0.0001 ≤ p < 0.001	337	0.1187	0.090%	1.32
0.00001 ≤ p < 0.0001	31	0.0109	0.009%	1.21
p < 0.00001	6	0.0021	0.001%	2.11

*Expected under uniform distribution. **Ratio of observed to expected.

Association results for 408 SNPs in 46 candidate genes (Table 4) revealed suggestive evidence for major CHD events for *ALOX5AP *(23 SNPs, 7 with p < 0.05 by GEE or FBAT), *GJA4 *(14 SNPs, 6 with p < 0.05), *MEF2A *(5 SNPs, 2 with p < 0.05), and *PCSK9 *(11 SNPs, 3 with p < 0.05). For HF, 4 SNPs in PLN and 2 each in ADRB2 and TPM1 had p values < 0.05. There was little evidence of association of AF with SNPs in specified candidate genes. Overall, 538 candidate-SNP association tests were carried out because there were 130 SNPs common to both major CHD and major CVD. Results with GEE p < 0.05 were obtained for 28 tests (5.2%) and p < 0.01 for 5 tests (0.9%), similar to the overall distribution in Table 3. Lack of consistency between GEE and FBAT results may be due to lower power of FBAT compared with GEE tests.

**Table 4:** Association Results for Pre-Specified Candidate Genes

Candidate gene	Total number of SNPs*	SNPs with p value < 0.05	Phenotype	GEE p value	FBAT p value
**Major CVD/Major CHD**					

*ALOX5*	5	0			
*ALOX5AP*	23	rs7983138	Major CHD	0.011	0.373
		rs2985183	Major CHD	0.014	0.455
		rs7984952	Major CHD	0.015	0.266
		rs117395	Major CHD	0.016	0.568
		rs4603405	Major CHD	0.018	0.257
		rs10507391	Major CHD	0.028	0.660
		rs10507391	Major CVD	0.043	0.878
		rs7995384	Major CHD	0.049	0.967
*GJA4*	14	rs618675	Major CHD	0.004	0.169
		rs10489658	Major CHD	0.004	0.145
		rs618675	Major CVD	0.009	0.464
		rs10493062	Major CHD	0.011	0.051
		rs768586	Major CHD	0.016	0.135
		rs10489658	Major CVD	0.025	0.237
		rs10489656	Major CHD	0.520	0.030
		rs10489656	Major CVD	0.538	0.044
		rs2093185	Major CVD	0.547	0.019
		rs6686484	Major CHD	1.000	0.031
*LGALS2*	1	0			
*LTA*	2	0			
*LTA4H*	22	rs10492225	Major CHD	0.013	0.080
*MEF2A*	5	rs2033546	Major CVD	0.004	0.006
		rs2863274	Major CVD	0.006	0.006
		rs2033546	Major CHD	0.016	0.013
		rs2863274	Major CHD	0.062	0.021
*MMP3*	17	rs2096767	Major CVD	0.028	0.506
		rs2096767	Major CHD	0.032	0.610
		rs566125	Major CVD	0.042	0.079
*SERPINE1*	2	0			
*PCSK9*	11	rs2114580	Major CHD	0.010	0.075
		rs2114580	Major CVD	0.026	0.057
		rs2317951	Major CVD	0.076	0.002
		rs2317951	Major CHD	0.077	0.002
		rs2317948	Major CHD	0.478	0.029
		rs2317948	Major CVD	0.584	0.026
*THBS2*	7	rs911839	Major CVD	0.192	0.035
		rs911839	Major CHD	0.255	0.032
*THBS4*	16	rs264986	Major CHD	0.443	0.048
*VAMP8*	5	0			

**Atrial fibrillation**					

*ACE*	3	0			
*AGT*	13	rs758216		0.041	0.204
*GJA5*	13	0			
*KCNE2*	14	0			
*KCNH2*	6	0			
*KCNJ2*	23	rs10512574		0.140	0.041
*KCNQ1*	5	rs10488674		0.136	0.046
*KCNE1*	20	rs7277304		0.745	0.047
		rs9305551		0.119	0.018

**Heart failure**					

*ABCC9*	8	0			
*ACTC*	15	rs752876		0.065	0.040
*ADRB1*	12	0			
*ADRB2*	18	rs40949		0.545	0.025
		rs185021		0.947	0.040
*ADRBK1*	0	-			
*ATP2A2*	3	0			
*CALML3*	2	0			
*CTF1*	0	-			
*DES*	2	0			
*DSP*	15	rs10484326		0.671	0.029
*LDB3*	0	-			
*LMNA*	5	0			
*MYBPC3*	4	0			
*MYH6*	1	0			
*MYH7*	2	0			
*MYL2*	3	0			
*MYL3*	1	0			
*PLN*	16	rs3951042		0.025	0.083
		rs724868		0.055	0.039
		rs9320660		0.063	0.034
		rs10484286		0.074	0.043
*SGCD*	37	0			
*TNNC1*	4	rs1133415		0.040	0.131
*TNNI3*	1	0			
*TNNT2*	9	rs832177		0.015	0.164
*TPM1*	7	rs10519186		0.011	0.085
		rs902027		0.152	0.011
*TTN*	13	rs10497521		0.705	0.030
*VCL*	3	0			

*Includes all SNPs within 200 kb upstream of start to 200 kb downstream of end of gene, with genotype call rate ≥0.8; minor allele frequency ≥0.1; HWE p ≥ 0.001.

Data are sorted by GEE additive genetic effects model with FBAT results provided alongside.

Additionally, we examined all association results for major CHD and major CVD in the region of chromosome 9 that was recently reported to be associated with MI and CHD [22,23], We found that 7 SNPs in a 76 Kb region had p < 10^-5 ^for one or both outcomes.

## Discussion

Cardiovascular disease is the leading cause of death in industrialized countries and will soon be the leading cause of death in the developing world [24]. Genome-wide association studies provide an opportunity to extend our understanding of CVD pathogenesis and improve public health. The identification of novel genes and pathways that play a causal role in CVD is an essential objective for the development of new therapies for the prevention and treatment of CVD. Finding genetic associations with CVD risk that are robust across multiple studies will aid in the personalization of medicine by identifying high risk individuals who can be targeted for early and aggressive preventive care.

We provide results of genome-wide association for 4 CVD outcomes of great public health impact: major CVD, major CHD, AF, and HF. No associations attained genome-wide significance [4.4 × 10^-8 ^= 0.05/(70,987 SNPs × 4 major traits × 2 adjustment levels × 2 association models)] in our analyses using GEE or FBAT additive genetic models. With dramatic declines in the cost of high throughput genotyping, selective genotyping of SNPs with suggestive evidence of association can be considered. Two-stage approaches – genome-wide association followed by selective genotyping – have been adopted as a practical and efficient strategy for pursuing initial genome-wide results [25,26].

Results of GEE and FBAT associations pointed to few candidate genes of obvious interest for any CVD outcomes. One intriguing result was the association of *RYR2 *(rs939698, p = 3.6 × 10^-4^) with HF. The ryanodine receptor has been implicated in arrhythmogenic right ventricular dysplasia/cardiomyopathy [21,27], a rare familial cardiomyopathy.

The lowest p values we identified may be purely by chance. The number of events (maximum of 142 for major CVD) was small to detect association, but would be sufficient to detect a SNP with high minor allele frequency in linkage disequilibrium with a causal variant that contributed high risk. This was the case for a genome-wide association study of age-related macular degeneration – only 96 cases and 50 controls were sufficient to identify genome-wide association with complement factor H [28]. Sometimes multiple SNPs in the same chromosomal region had low GEE p values for a trait; for example, Table 2a has SNP clusters on chromosomes 6, 9, 11, 13, 15 and 17. Linkage disequilibrium exists for those clustered SNPs (typically, pair-wise r^2 ^above 0.80) and it is uncertain whether the concordant results represent statistically correlated chance findings or indicate regions of heightened interest.

Candidate gene results for the 4 CVD outcomes provided suggestive confirmation of prior associations reported for *ALOX5AP *(23 SNPs, 7 with p < 0.05 by GEE or FBAT), *GJA4 *(14 SNPs, 6 with p < 0.05), *MEF2A *(5 SNPs, 2 with p < 0.05), and *PCSK9 *(11 SNPs, 3 with p < 0.05) in relation to CHD risk. In contrast, candidate gene results for HF and AF provided little evidence of replication of previously reported associations. Null results of these associations may be due in part to poor coverage of the candidates by the SNPs on the 100K chip and the modest number of events available for analysis. Our results can be compared with other genome-wide associations of similar phenotypes. We observed strong association of major CVD with 3 SNPs in the region of chromosome 9 that was recently reported to be associated with MI and CHD in multiple samples [22,23]. This provides convincing evidence that, despite modest numbers of events, we were able to identify true associations.

This investigation has several limitations. This study used CVD cases that were identified through careful surveillance of a community-based sample with multigenerational participation. Recruitment of original and offspring cohort participants began long before DNA collection, which occurred in recent years. Thus, most CVD cases were prevalent at the time of DNA collection. For CVD outcomes (such as these) with substantial mortality risk, a survival bias may have been introduced by this study design; individuals with early CVD events had to survive and attend a later clinic examination at which DNA was collected. Another limitation is the modest number of events included in analyses, in particular for HF, where only 73 events were available for analysis. For continuous traits, we had 78% power to detect a SNP with QTL heritability of 1% at significance level 10^-3^, and at significance level 10^-6 ^we had 84% power for QTL heritability 2% [20]. In the setting of a limited number of outcome events, those are large effect sizes. The negative results of candidate gene analyses may underestimate associations for genes that are incompletely covered by the SNPs used in this investigation. Lastly, a large proportion of the results are likely to be due to chance. Replication studies are needed to determine which, if any, of the results we report are indicative of true associations of causal variants with disease outcomes.

These association results for major CVD outcomes extend experience with genome-wide association studies. Replication studies are needed and will be used to guide future genotyping and resequencing efforts. Finding genetic variants associated with CVD may facilitate the identification of high risk patients and aid in identifying targeted future approaches to prevention and treatment of CVD.

## Abbreviations

AF = atrial fibrillation; CHD = coronary heart disease; HF = heart failure; CVD = cardiovascular disease; FBAT = family based association test; GEE = generalized estimating equation.

## Competing interests

The authors declare that they have no competing interests.

## Authors' contributions

MGL participated in study design, data collection, statistical analysis, interpretation of results, and manuscript preparation. LDA contributed to the design and analysis. EJB contributed to the design of analyses, acquisition and interpretation of data, provided critical manuscript revisions. LAC contributed to the design and analysis. RBD contributed to the design and analysis. CSF contributed to data acquisition and approved the final version of the manuscript. DRG contributed to project design and data acquisition. CYG participated in statistical analysis. NLHC contributed to the design and analysis. SJH participated in statistical analysis and manuscript preparation. JMM participated in acquisition of data, interpretation, revising & approval of final manuscript. CNC participated in the analysis and interpretation of data and critical review of the manuscript. CJOD participated in the analysis and interpretation of data and critical review of the manuscript. SS participated in data collection, definition of phenotypes and review of the manuscript. RSV participated in data collection, interpretation of analyses and review of the manuscript. TJW contributed to data acquisition data, interpretation of data analysis, and revision of the manuscript for important intellectual content. PAW participated in data collection and project conception and design. DL contributed to project conception and design, interpretation of results, and drafting the manuscript.

All authors approved the final manuscript.
